# Novel Poxvirus in Proliferative Lesions of Wild Rodents in East Central Texas, USA

**DOI:** 10.3201/eid2406.172057

**Published:** 2018-06

**Authors:** Carolyn L. Hodo, Matthew R. Mauldin, Jessica E. Light, Kimberly Wilkins, Shiyuyun Tang, Yoshinori Nakazawa, Ginny L. Emerson, Jana M. Ritter, Joanne L. Mansell, Sarah A. Hamer

**Affiliations:** Texas A&M University, College Station, Texas, USA (C.L. Hodo, J.E. Light, J.L. Mansell, S.A. Hamer);; Centers for Disease Control and Prevention, Atlanta, Georgia, USA (M.R. Mauldin, K. Wilkins, S. Tang, Y. Nakazawa, G.L. Emerson, J.M. Ritter)

**Keywords:** *Chordopoxvirinae*, histopathology, microscopy, electron microscopy, phylogenetics, *Poxviridae*, *Chordopoxvirinae*, *Baiomys taylori*, infections, viruses, poxvirus, zoonoses, rodents, northern pygmy mouse, epidermal lesions, proliferative lesions, East-Central Texas, United States

## Abstract

Northern pygmy mice from 2 localities in East Central Texas, USA, had proliferative epidermal lesions on the tail and feet. Electron microscopy of lesion tissue revealed poxvirus. Phylogenetic analyses indicated the virus differed 35% from its closest relatives, the *Chordopoxvirinae*. Future research is needed to determine whether this virus could affect human health.

*Chordopoxvirinae* is a diverse subfamily of viruses within *Poxviridae*. These geographically widespread viruses infect birds, reptiles, and mammals, and many are zoonotic (*1*). The increasing use of molecular methods has resulted in the identification of several novel poxviruses from humans and animals, many of which probably represent new genera (*2*–*9*); often, the reported host range of these viruses is limited to the species of the index case. Novel human poxvirus infections identified in the 21st century have often been presumed to have animal origins; for example, an investigation of a novel poxvirus isolated from 2 men in the country of Georgia revealed serologic evidence of orthopoxvirus exposure in cows in their herd and in captured rodents (*5*). We report a novel poxvirus infection characterized by proliferative epidermal lesions in wild northern pygmy mice (*Baiomys taylori*) found at 2 localities in east central Texas, USA, and further characterize the virus through genetic analysis.

## The Study

**Figure 1:**
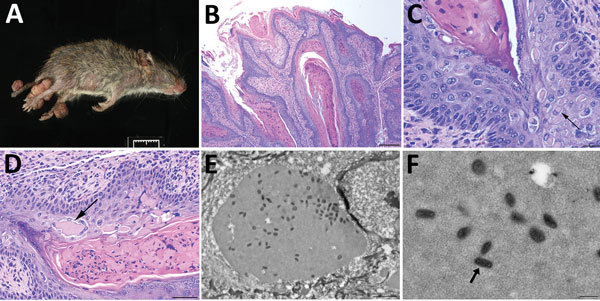
Histologic analysis and electron microscopy of lesions from *Baiomys taylori* mouse 1 infected with novel poxvirus, east central Texas, USA, 2014. A) Large epidermal masses arose from skin of both hind limbs and tail. B–D) Skin mass. Hematoxylin and eosin stain. B) The proliferative epidermis forms papillary projections with abundant hyperkeratosis and thickening of the stratum spinosum and stratum granulosum. Scale bar indicates 200 µm. C, D) Keratinocytes contain intracytoplasmic eosinophilic viral inclusions (C, arrow) that occasionally form extracellular lakes (D, arrow). Panel C scale bar indicates 20 µm; panel D scale bar indicates 50 µm. E, F) Electron microscopy. E) Cytoplasmic inclusions containing granular, electron-dense material and numerous brick-shaped virions. Scale bar indicates 1.5 µm. F) Virions have dense cores and shells of inner and outer membranes consistent with poxvirus (arrow). Scale bar indicates 300 nm.

In August 2014, we captured a severely affected adult male *B. taylori* mouse (mouse 1) at the Attwater Prairie Chicken National Wildlife Refuge in Colorado County, Texas. The mouse had large (4–8-mm diameter) proliferative lesions on the hind feet and tail (Figure 1, panel A) but otherwise appeared healthy. In April 2017, at the Biodiversity Research and Teaching Collections at Texas A&M University in College Station, Texas (160 km north of the first locality), we captured an additional adult male *B. taylori* mouse (mouse 2) with mild 1–2-mm proliferative lesions on the left hind foot and tail. Both animals were euthanized in accordance with Texas Parks and Wildlife Department scientific collections permit (SPR-0512-917) and Texas A&M University Institutional Animal Care and Use Committee’s animal use protocol (2015-0088). These 2 *B. taylori* specimens are housed at Biodiversity Research and Teaching Collections (mammal voucher nos. TCWC 65223 and TCWC 65224; http://portal.vertnet.org/search).

We subjected mouse 1 to a full necropsy and found the mouse to be in good body condition. Extending from the skin of the dorsal aspect of the left hind foot, plantar aspect of the right hind foot, and the dorsal tail were several firm, pedunculated, irregular masses 0.4–0.8 cm in diameter (Figure 1, panel A). When the masses were sectioned, the cut surface was light tan with a papillated appearance, and the masses did not appear to invade the underlying tissues. The tail was partially amputated but was healed and apparently unrelated to the lesions. We did not observe any other lesions. We froze a section of a mass at −80°C for molecular work and fixed the remaining tissues in 10% neutral-buffered formalin; the fixed tissues were processed for histology and stained with hematoxylin and eosin.

On microscopic examination, necrotizing and proliferative dermatitis was observed. The epidermis of the affected area had multiple large, exophytic, pedunculated masses composed of markedly hyperplastic epithelial cells forming papillary projections with abundant orthokeratotic and parakeratotic hyperkeratosis (Figure 1, panel B). The epithelial surface was multifocally eroded to ulcerated, and the stratum corneum contained aggregates of coccoid bacteria. The stratum spinosum and stratum granulosum were markedly thickened with swollen keratinocytes (ballooning degeneration) frequently containing intracytoplasmic eosinophilic viral inclusions (Figure 1, panels B, C). These inclusions frequently extended extracellularly, forming large lakes <50 μm in diameter. The dermis and stratum basale were infiltrated by lymphocytes, plasma cells, and macrophages and a large number of viable and degenerate neutrophils. We examined sections of the spleen, liver, lungs, heart, kidneys, and intestines and identified no substantial lesions. A viral etiology was suspected, so formalin-fixed paraffin-embedded sections of the mass were processed for transmission electron microscopy. Ultra-thin sections were examined with a Morgagni 268 transmission electron microscope (FEI, Hillsboro, OR, USA) at an accelerating voltage of 80 kV. Cytoplasmic inclusions contained granular, electron-dense material and numerous brick-shaped virions with dense cores and inner and outer membrane shell consistent with poxvirus (Figure 1, panel D).

**Figure 2:**
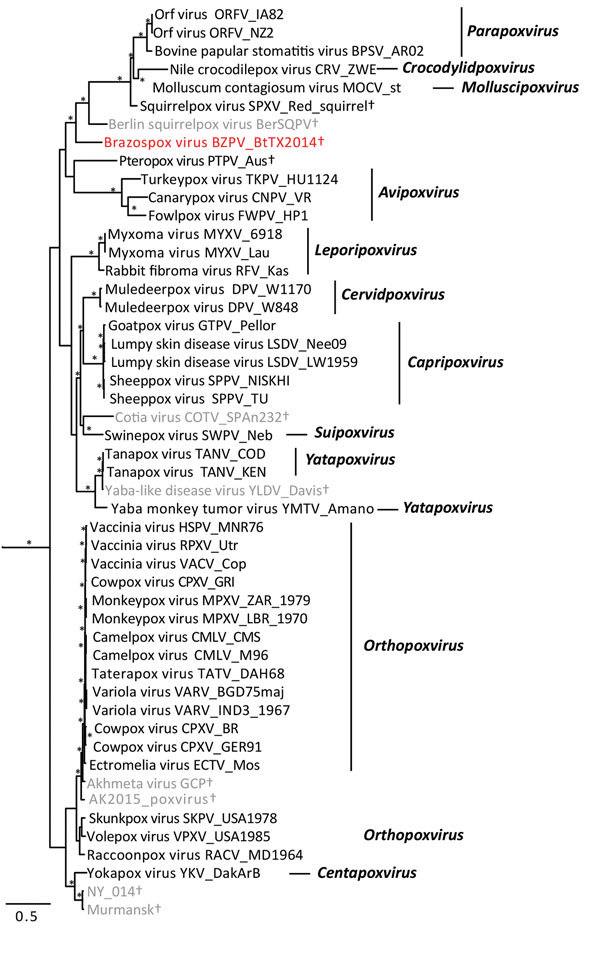
Bayesian phylogram of poxvirus isolates constructed by using a concatenated 27,674-bp alignment of 9 conserved open reading frames (Copenhagen homologs A7L, A10L, A24R, D1R, D5R, E6R, E9L, H4L, and J6R). Genera (if assigned) are listed. Brazospox virus (red; GenBank accession nos. MG367480–8) and sequences from other species not recognized by the International Committee on Taxonomy of Viruses (gray) are indicated. *Nodes with posterior probabilities >0.95; †species not assigned to a genus. Scale bar corresponds to the number of nucleotides substitutions per site.

We extracted DNA from skin masses of both mouse 1 and mouse 2 and subjected the DNA to PCR with low–GC content poxvirus primers targeting a region of the putative metalloproteinase gene (*10*) and Sanger sequencing. The virus sequences (220 bp) from mouse 1 (BtTX2014) and mouse 2 (BtTX2017) were identical to each other (GenBank accession no. MG367479), and the top 10 matches to this sequence in GenBank were all poxviruses with only 76%–78% shared identity. In addition, we extracted DNA from formalin-fixed paraffin-embedded sections of liver, lung, kidney, and spleen from mouse 1, and all were PCR negative for poxvirus DNA. A DNA aliquot of the mass from mouse 1 was sent to Otogenetics (Atlanta, GA, USA) for whole-genome sequencing with an Illumina HiSeq platform (Illumina Inc., San Diego, CA, USA). From these data, we extracted 9 core genes of BtTX2014 located within the conserved coding portion of the poxvirus genome used in previous studies (*5*) (GenBank accession nos. MG367480–8). In Geneious version 8.1.4 (https://www.geneious.com/), we converted the gene sequences into amino acid sequences for the purpose of alignment to those of 49 other chordopoxviruses (*Chordopoxvirinae*) and 2 entomopoxviruses (*Entomopxvirinae*), which were used as outgroup taxa. We then concatenated the gene sequences (total alignment 27,674 bp) and conducted partitioned phylogenetic analyses using Bayesian approaches in MrBayes version 3.2.2 (http://mrbayes.sourceforge.net/download.php). The phylogenetic position of this virus revealed it to be a divergent member of the *Chordopoxvirinae* (Figure 2), divergent from other *Chordopoxvirinae* poxviruses by an average of 35% (uncorrected p distances for the 27,674 bp of the concatenated alignment; Technical Appendix Table).

## Conclusions

The poxvirus sequenced from *B. taylori* in east central Texas is distinct from previously identified viruses, with genetic distances similar to those observed between genera (online Technical Appendix Table). Although support for several phylogenetic relationships is low (possibly due to high genetic variation within *Chordopoxvirinae*), the genetic data strongly suggest this poxvirus does not belong to any recognized genus as of March 2018. We propose the tentative species name Brazospox virus in reference to the proximity of both field sites to the Brazos River.

The epidermal lesions produced by the virus are unique among previously described poxviruses of wild rodents, being proliferative rather than ulcerative in nature, and lacking systemic involvement (*7*,*11*). The population-level implications of this poxvirus on hosts are unclear. The 2 mice in this study represent the spectrum of observed pathology, from severe (mouse 1) to mild (mouse 2). The virus was confirmed at 2 different localities, and field notes indicate similar lesions were observed in other rodent species. During 2013–2017 (*12*,*13*), among ≈1,800 rodents captured during field research in east central Texas, we documented proliferative lesions on the tail or feet of >17 individual rodents of 3 species (*B. taylori*, n = 12; *Chaetodipus hispidus*, n = 2; *Sigmodon hispidus*, n = 3). The combined distributional range of these 3 host species includes >20 US states and a large portion of Mexico (*14*).

Novel poxviruses identified in wildlife populations might be useful for the identification of threats to human and animal health. The description of these new viruses contributes to the study of viral diversity and pathogenesis. Propagation of the Brazospox virus in cell culture for use in infection studies, coupled with expanded field surveillance, examination of museum specimens, and full genome analysis could yield additional clues to the origin, pathogenesis, and potential host range of this novel poxvirus.

Technical Appendix Taxon information and genetic distances of poxviruses used in phylogenetic analysis of novel Brazospox virus isolate.
