# Supplementary material for: Novel Poxvirus in Proliferative Lesions of Wild Rodents in East Central Texas, USA
Source: Emerg Infect Dis. 2018 Jun;24(6):1069–72. doi: 10.3201/eid2406.172057 (PMC6004845; doi:10.3201/eid2406.172057)
Supplement: Technical Appendix — Taxon information and genetic distances of poxviruses used in phylogenetic analysis of novel Brazospox virus isolate. [file 17-2057-Techapp-s1.pdf]

Article DOI: https://doi.org/10.3201/eid2406.172057

Novel Poxvirus in Proliferative Lesions of Wild Rodents in East-Central Texas, USA

Technical Appendix

Technical Appendix Table. Taxon information and genetic distances of poxvirus isolates included in phylogenetic analysis of Brazospox virus BtTX2014 sequence, East-Central Texas, 2014

Species*	Genus	Isolate†	GenBank accession no.	Genetic distance from Brazospox virus, %‡
Brazospox virus§	Unassigned	BtTX2014	MG367480–8	
Canarypox virus	*Avipoxvirus*	CNPV_VR	NC_005309	38.2
Fowlpox virus	*Avipoxvirus*	FWPV_HP1	AJ581527	38.9
Turkeypox virus	*Avipoxvirus*	TKPV_HU1124	NC_028238	39.1
Goatpox virus	*Capripoxvirus*	GTPV_Pellor	NC_004003	33.6
Lumpy skin disease virus	*Capripoxvirus*	LSDV_LW1959	AF409138	33.5
Lumpy skin disease virus	*Capripoxvirus*	LSDV_Nee09	NC_003027	33.5
Sheeppox virus	*Capripoxvirus*	SPPV_NISKHI	AY077834	33.5
Sheeppox virus	*Capripoxvirus*	SPPV_TU	NC_004002	33.5
Yokapox virus	*Centapoxvirus*	YKV_DakArB	HQ849551	33.4
Muledeerpox virus	*Cervidpoxvirus*	DPV_W1170	AY689437	32.9
Muledeerpox virus	*Cervidpoxvirus*	DPV_W848	NC_006966	32.8
Nile crocodilepox virus	*Crocodylidpoxvirus*	CRV_ZWE	NC_008030	48.3
Myxoma virus	*Leporipoxvirus*	MYXV_6918	EU552530	34.8
Myxoma virus	*Leporipoxvirus*	MYXV_Lau	NC_001132	34.8
Rabbit fibroma virus	*Leporipoxvirus*	RFV_Kas	NC_001266	34.6
Molluscum contagiosum virus	*Molluscipoxvirus*	MOCV_st	NC_001731	43.4
Camelpox virus	*Orthopoxvirus*	CMLV_CMS	AY009089	33.0
Camelpox virus	*Orthopoxvirus*	CMLV_M96	NC003391	33.0
Cowpox virus	*Orthopoxvirus*	CPXV_BR	NC_003663	33.0
Cowpox virus	*Orthopoxvirus*	CPXV_GER91	DQ437593	33.0
Cowpox virus	*Orthopoxvirus*	CPXV_GRI	X94355	32.8
Ectromelia virus	*Orthopoxvirus*	ECTV_Mos	NC004105	33.1
Monkeypox virus	*Orthopoxvirus*	MPXV_LBR_1970	DQ011156	33.0
Monkeypox virus	*Orthopoxvirus*	MPXV_ZAR_1979	DQ011155	33.0
Raccoonpox virus	*Orthopoxvirus*	RACV_MD1964	FJ807746–54	33.1
Skunkpox virus	*Orthopoxvirus*	SKPV_USA1978	FJ807755–63	32.8
Taterapox virus	*Orthopoxvirus*	TATV_DAH68	NC008291	33.0
Vaccinia virus (horsepox)	*Orthopoxvirus*	HSPV_MNR76	DQ792504	32.9
Vaccinia virus (rabbitpox)	*Orthopoxvirus*	RPXV_Utr	AY484669	32.9
Vaccinia virus	*Orthopoxvirus*	VACV_Cop	M35027	32.9
Variola virus	*Orthopoxvirus*	VARV_BGD75maj	L22579	33.0
Variola virus	*Orthopoxvirus*	VARV_IND3_1967	NC_001611	33.1
Volepox virus	*Orthopoxvirus*	VPXV_USA1985	FJ807737–45	33.0
Bovine papular stomatitis virus	*Parapoxvirus*	BPSV_AR02	NC_005337	45.2
Orf virus	*Parapoxvirus*	ORFV_IA82	AY386263	45.4
Orf virus	*Parapoxvirus*	ORFV_NZ2	DQ184476	45.4
Swinepox virus	*Suipoxvirus*	SWPV_Neb	NC_003389	33.2
Akhemta virus§	Unassigned	GCP	KM046934–42	33.1
Cotia virus§	Unassigned	COTV-SPAn232	HQ647181	34.8
Pteropox virus	Unassigned	PTPV_Aus	KU980965	36.9
Squirrelpox virus	Unassigned	SPXV_Red_squirrel	HE601899	41.0
Yaba-like disease virus§	Unassigned	YLDV_Davis	NC_002642	33.2
AK2015_poxvirus§	Unassigned	AK2015_poxvirus	KX914668–76	33.0
NY_014§	Unassigned	NY_014	MF001305	32.8
Murmansk§	Unassigned	LEIV-11411 Mur-Lovozero	MF001304	32.8
Berlin squirrelpox virus (BerSQPV)§	Unassigned	BerSQPV	MF503315	31.4
Tanapox virus	*Yatapoxvirus*	TANV_COD	EF420157	33.3
Tanapox virus	*Yatapoxvirus*	TANV_KEN	NC_098882	33.3

Species*	Genus	Isolate†	GenBank accession no.	Genetic distance from Brazospox virus, %‡
Yaba monkey tumor virus	*Yatapoxvirus*	YMTV_Amano	NC_005179	33.4
Amscata moorei entomopoxvirus L	*Betaentomopoxvirus*	AMEV_Moyer	NC_002520	60.0
Melanoplus sanguinipes entomopoxvirus O	Unassigned	MSEV_Tucson	NC_001993	60.3

*Species names follow the International Committee on Taxonomy of Viruses, although not all isolates have been officially recognized as species and some are unassigned to a genus. All isolates belong to the subfamily *Chordopoxvirinae*, except for the outgroup taxa (AMEV_Moyer and MSEV_Tuscon), which belong to the *Entomopoxvirna.*
†Isolates match taxon labels in Figure 2 of main text.
‡Genetic distances are uncorrected p distances.
§Not officially recognized species.
